# Role of transcription factor Sp1 and CpG methylation on the regulation of the human podocalyxin gene promoter

**DOI:** 10.1186/1471-2199-7-17

**Published:** 2006-05-09

**Authors:** Nora Butta, Susana Larrucea, Sonia Alonso, Ramón B Rodriguez, Elena G Arias-Salgado, Matilde S Ayuso, Consuelo González-Manchón, Roberto Parrilla

**Affiliations:** 1 Department of Physiopathology and Human Molecular Genetics Centro de Investigaciones Biologicas (CSIC), Ramiro de Maeztu 9 28040-Madrid, Spain

## Abstract

**Background:**

Podocalyxin (podxl) is a heavily glycosylated transmembrane protein mainly found on the apical membrane of rat podocytes and also in endothelial, hematopoietic, and tumor cells. Despite of its interest no much is known about the transcriptional regulation of podxl in different cells. Thus, we aimed at studying the functional features of the 5'-regulatory region of the human Podxl gene.

**Results:**

The promoter region of the human Podxl gene has been cloned and its structure and function were analyzed. The primary DNA sequence is rich in G+C and is devoid of TATA or CAAT boxes. The sequence contains recognition sites for several putative transcription factors; however, the basic promoter activity seems to rely entirely on Sp1 transcription factor since supershift analysis was positive only for this factor. The region encompassed by 66 to -111 nts conferred the minimal transcriptional activity that increases as the number of Sp1 sites augmented with the length of the promoter fragment. In Sp1-lacking insect cells the Podxl promoter constructs showed activity only if cotransfected with an Sp1 expression plasmid. Finally, mutation of the Sp1 sites reduced the promoter activity.

We analyzed whether methylation of the CpG dinucleotides present in the first ~600 nts of the promoter region of Podxl could explain the variable rates of expression in different types of cells. Inactivation of methyltransferases by 5'-aza-2'deoxicitidine showed a dose-dependent increase in the podxl content. Moreover, in vitro methylation of the promoter constructs -111,-181 and -210 led to an almost complete reduction of the promoter activity. A correlation was found between the degree of methylation of the CpG promoter dinucleotides and the rate of podxl expression in different cell lines.

**Conclusion:**

Our results indicate that transcriptional regulation of Podxl is supported primarily by Sp1 site(s) and that DNA-methylation of the CpG promoter islands contributes to control the tissue specific expression of podxl.

## Background

Podocalyxin (podxl) is an integral membrane protein originally identified by Kerjaschki et al [1] in the foot processes of the rat kidney glomerular podocytes. It was described as a sialoprotein of 140-kDa based on its staining with alcian blue. The high sulfate content of this protein contributes in part to its heavily negative charge [2] that is supposed to maintain open the interdigitations of the podocyte foot for the urine filtration. The importance of the negative charge of this protein is indicated by the loss of the structure and function of the podocytes by treatment with polycations or desialylation with neuraminidase [3,4]. Null mice for podxl die soon after birth due to failure to develop foot processes in the glomerular epithelial cells [5]. Podxl like proteins have been cloned from the rabbit, human, chicken and mouse [6-9].

The podxl is also expressed in the luminal face of the vascular endothelia, hematopoietic cells and platelets [10,11], among other tissues. The function of this protein in non-renal cells has not yet been elucidated. It has been suggested that it may function as a ligand for leucocyte adhesion molecule L-selectin [12] due to its location in the postcapillary venules of the lymph node, high endothelial venules, and the fact that most of the L-selectin ligands in the vascular endothelium are sialomucins. Thus, the podxl of the vascular endothelia may contribute to the migration of lymphocytes toward the lymph nodes.

The podxl proteins show a poorly conserved aminoterminal, extracellular, mucin-like domain containing multiple *N- *and *O*-linked glycosylation sites, a disulfide containing domain and conserved transmembrane and cytoplasmic domains. The cytoplasmic tails showed the highest identities among these proteins and interaction with cytosolic proteins have been reported. Based on structural similarities and their high content in sialic acid, Sassetti et al [13] proposed that CD34, podxl and endoglycan, would form a family named the CD34 family of sialomucins. The three proteins are found in the vascular endothelia and also in early hematopoietic precursors, suggesting that their function may go from either cell anti-adherence, as it is the case in the renal podocytes, to selective adherence or control of cellular differentiation.

Despite the interest of podxl no much is known about the transcriptional regulation of its gene in different cells. The binding of the Wilms Tumor suppressor factor (WT1) to conserved elements within the Podxl gene promoter results in a potent transcriptional activation [14] that seems to be relevant only for the induction and development of the kidney; however, a detailed analysis of the human Podxl promoter (Podxl-pr) has not yet been performed. In the present study we have further characterized the 5' regulatory region of this gene. We found absence of a TATA-box and a transcriptional regulation supported primarily by Sp1 site(s). In addition, the role of cytosine methylation of the promoter region has been analyzed to elucidate whether this epigenetic mechanism might contribute to control the specific regulation of the expression of this gene.

## Results

### Features of the 5' flanking region of the human podocalyxin

The human Podxl gene and 250 bps upstream of the putative start methionine was previously cloned and mapped to chromosome 7q32-q33 by Kershaw et al [7,15]. We amplified by PCR a 1528 bp-DNA fragment of the 5' regulatory region of the human Podxl gene comprising 1297 bp from the transcription start site plus 231 bp of 5'-untranslated region, using the primers sense: -1297/-1275 and antisense: 231/208. We started numbering from the transcription start site [GenBank:AF395890]. Computer analysis using the software "Transcription Factor Binding Site Profile Database" (TFSEARCH program) [16], revealed a high G+C content of the proximal promoter region and a lack of CCAAT and TATA-like elements, a feature commonly found in the house-keeping genes. We also detected potential binding sites for the transcription factors AP2 (-247/-236 and -114/-93 nts) (two overlapped sites); overlapped Sp1 sites (167/-140, -146/-128 and -91/-68 nts); overlapped Sp1 and AP2 sites (-279/-266 nts), overlapped Sp1 and NFKB sites (-204/-189 nts), three GATA-1 sites (-1138/-1129, -1057/-1051 and -444/-435 nts) and three WT-1 sites (two overlapped at -1233/-1216 and one at -507/-498 nts).

**Figure 1:**
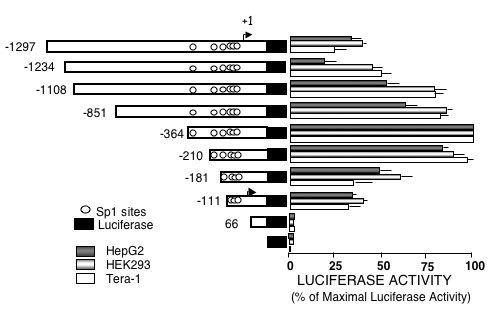
**Transient transfection of deletion mutants of the 5'-flanking region of the human podocalyxin gene**. Description of the construction of the pGL3-Podxl-pr reporter plasmids and transient transfection experiments are described under "Methods". Luciferase activity was corrected for transfection efficiency with the values obtained with Renilla and was expressed as percent of the maximal activity of the -364 construct. The results are mean ± SEM of three experiments performed in duplicates.

**Figure 2:**
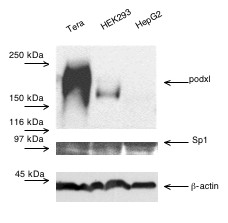
**Western blots of human podocalyxin and Sp1 transcription factor**. Cell lysates (20 μg) were resolved on SDS-7.5% polyacrylamide gels under reducing conditions, transferred to PVDF membranes and immunoblotted with either 1 μg/ml of anti-podxl or anti Sp1 antibodies. β-Actin content was blotted as a control of gel loading. The experiment was repeated three times.

To determine the location of the promoter activity we studied the transcriptional activity of chimeric constructs of progressively 5'-deleted DNA fragment (-1297/231) linked to the luciferase (pGL3) reporter (Fig. 1). The constructs were transiently transfected into Tera-1, HEK293 and HepG2 cells, which podxl content differs considerably (Fig. 2). Figure 1 depicts the results obtained with the three types of cells. No significant transcriptional activity was detected with the fragment of +66 bp. The activity started on construct -111, increasing with the length of the fragment up to -364. Thus, the basal promoter activity is located within the -111 to +66 bp region, whereas the upstream 253 bp confers maximal transcriptional activity. The extension of the 5' flanking sequence up to -1297 diminished the promoter activity. All cells expressed similar amounts of Sp1 (Fig. 2, middle row).

### Regulatory role of Sp1 on Podxl promoter activity

**Figure 3:**
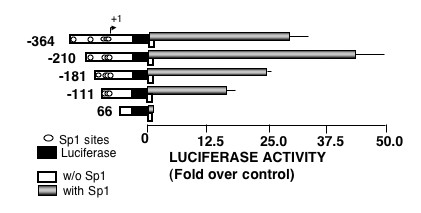
**Effect of Sp1 on podocalyxin gene promoter activity in insect cells**. *Drosophila*-derived SL2 cells were transiently cotransfected with 5 μg of pGL3/Podxl-pr constructs and with either 0.5 μg of pPAC void plasmid or 0.5 μg of pPAC-Sp1 plasmid. Luciferase activities were normalized by protein concentration and are shown as the fold increases relative to the basal activity of void pGL3-basic plasmid (control). Values reported in the figure are mean ± SEM of three independent experiments in duplicates.

The fragments up to the first 364 bp show a progressive increasing number of Sp1 sites, suggesting their role in transcription. To test this possibility we transfected some of the constructs into the Sp1-deficient Drosophila-derived Schneider's SL2 cells. As indicated in Fig. 3, no transcriptional activity could be detected when the constructs +66, -111, -181,-210 and -364 were transfected into these cells; however, the transcriptional activity was initiated in all the constructs, except for the +66, when the cells were cotransfected with a plasmid expressing Sp1 (Fig. 3). Thus, the Sp1sites seem to be essential to maintain a normal basal rate of transcription of Podxl.

**Figure 4:**
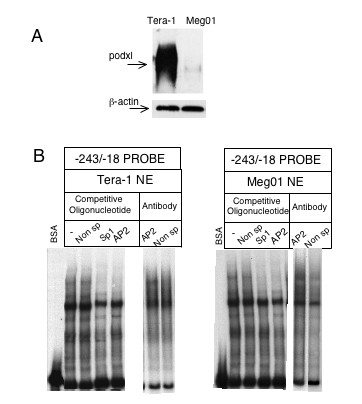
**Gel mobility-shift analysis of DNA-protein complexes using the human podocalyxin promoter fragment -243/-18**. A) Western blots were performed as described under Fig. 2; B) Five μg of nuclear extracts (NE) from Tera-1 and Meg0l cells were incubated with the probe. Competitive gel shift assay was done in the presence of 100-fold molar excess of unlabeled double-stranded oligonucleotides corresponding to the pointed out transcription factors consensus and unrelated nonspecific (Non Sp) motifs. Antibodies (2 μg) for AP2 or non-specific were preincubated with the Tera-1 and Meg0l NE before the addition of the Podxl probe.

The DNA/protein interactions were characterized by electrophoretic mobility of DNA-protein complexes (EMSA). The assays were performed with two different probes encompassing the -243/-18 and -538/-238 Podxl fragments and nuclear extracts from cells with either low or high levels of podxl expression (Meg0l and Tera-1 cells, respectively, Fig. 4A). Nuclear extracts from Meg0l and Tera-1 cells formed retardation complexes with the -243/-18 and -538/-238 probes. The identity of proteins interacting at the potential binding sites was assessed using competitor oligonucleotides for AP2, Sp1, GATA-1 and NFκB sites and a non-specific unrelated oligonucleotide. AP2 as well as Sp1 oligonucleotides prevented the formation of the same protein/DNA complex within the -243/-18 probe (Fig. 4B), whereas no effect was observed (data not shown) with any of the other oligonucleotides. No displacement of the EMSA bands was appreciated with an antibody against AP2, regardless of the type of nuclear extract used (Fig. 4B). With the -538/-238 probe no competition was observed with a 100-fold excess of oligonucleotides (data not shown).

**Figure 5:**
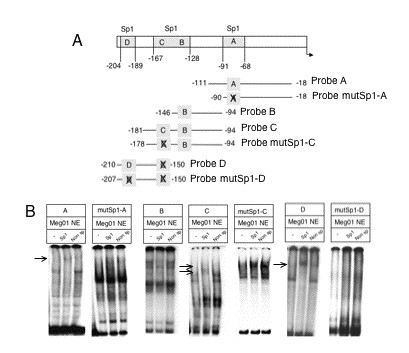
**Identification of Sp1-DNA complexes by supershift assays**. A) Different probes were designed as described under "Methods" in order to identify functional Sp1 sites in the Podxl promoter. B) Antibodies (2 μg) for Sp1 and non-specific, were preincubated with 5 μg of Meg01 nuclear extracts (NE) before the addition of the wild or mutated Sp1-containing Podxl probes. BSA (2 μg) was used as a control. An arrow points to the supershifted Sp1 bands.

**Figure 6:**
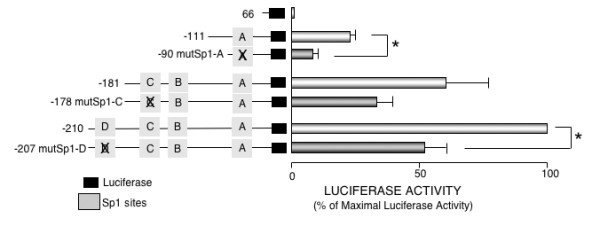
**Effect of mutating the Sp1 recognition sites on the activity of podocalyxin promoter**. Transient transfections were performed into HEK293 cells. Results represent mean ± SEM from four independent experiments performed in duplicates. Promoter activity of each transfection was expressed as a ratio of luciferase/renilla activities. Data are expressed as percent of the maximal activity of the -210 construct *p < 0.05 vs. non Sp1-site-mutated construct.

We ran supershift assays using nuclear extracts from Meg0l cells on different probes: "A" probe includes -91/-68 overlapped Sp1 sites (Sp1-A); "B" probe includes -146/-128 Sp1 sites (Sp1-B); "C" probe includes -146/-128 and -167/-140 sites (Sp1-B and Sp1-C), and "D" probe that only includes -204/-189 Sp1 site (Sp1-D, Fig. 5A). As shown in Fig. 5B, protein-DNA complexes were formed with all the probes tested and at least one of the complexes was shifted by anti-Sp1, excepting those in which probe B were used. Therefore, the binding of nuclear proteins to the Sp1-C site may explain the supershifted complexes observed with "C" probe. When probes with mutated Sp1 sites were used no supershifts were observed (Fig. 5B). Similar results were obtained when Tera-1 nuclear extracts were used (data not shown). In agreement with the EMSA assays, mutations in either Sp1-A, Sp1-C or Sp1-D sites inhibited the activity of the Podxl promoter by ~50% (Fig. 6) whereas mutation of Sp1-B did not (data not shown).

### Effect of promoter CpG methylation on the cellular expression of podxl

**Figure 7:**
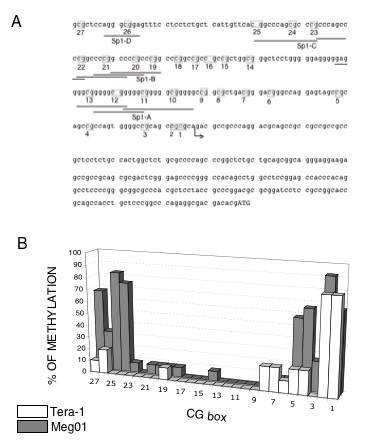
**State of methylation of CpG dinucleotides in the human podocalyxin promoter**. A) Schematic representation of a fragment of the Podxl-pr comprising 208 nts upstream the transcription start site (indicated with an arrow) and the 5'UTR. Grey boxes denote the positions of CpG dinucleotides. Underlined nts indicate Sp1 recognition sites. B) Genomic DNA from Tera-1 or Meg0l cells were extracted and subjected to bisulfite treatment, PCR amplification and DNA sequencing as described in Methods. CpG sites are numbered from 1 to 27 relative to the transcription start site. Three bisulfite treatments were performed. Data is expressed as % of methylation, calculated as percentage of conserved C in at least 10 clones obtained after each bisulfite treatment.

DNA methylation has been suggested to play a role in maintaining the stability of cell differentiation and in regulating gene transcription [17,18]. It has been recently reported that the selective demethylation of a promoter DNA may be a general mechanism required for the reprogramming of somatic cell nuclei [19]. DNA sequences rich in CpG are susceptible of methylation and therefore silencing the gene expression. Computer analysis of the 5'-flanking region of the Podxl gene with CpGPlot program [20] shows the existence of a putative CpG island, from -607 to the translation start site, containing 27 CpG dinucleotide sequences. The methylation of cytosines may control the transcriptional activity through changes in the binding activity of Sp1 sites. Thus, in order to elucidate the possible epigenetic regulation of Podxl we analyzed the methylation status of individual promoter CpG sites in cells expressing either high (Tera-1) or low (Meg0l) levels of podxl (Fig. 4A). Genomic DNA of both cell lines were treated with bisulfite and then amplified by PCR with the specific primers described under Methods. Bisulfite changes all the unmethylated cytosines to uracils and the uracils are PCR-amplified as thymidines, whereas the methylated cytosines are not modified. As indicated in Fig. 7, Tera-1 and Meg0l cells showed different patterns of methylation. In high-podxl expressing cells the promoter region is unmethylated whereas in low-podxl expressing cells, Meg0l cells, the promoter is partially methylated. The main difference was observed in the methylation of CpG boxes from 23 to 27 (Fig. 7).

**Figure 8:**
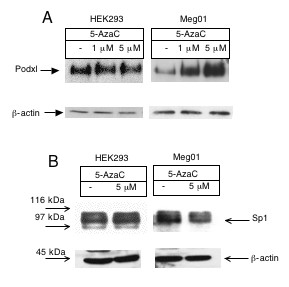
**Expression of human podocalyxin in HEK293 and Meg0l cells treated with 5-AzaC**. Cells were treated with 5-AzaC for 48 hrs. Cell lysates were resolved on SDS-7.5% polyacrylamide gels under non-reducing conditions, transferred to PVDF membranes and immunoblotted with A) 1 μg/ml of anti-podocalyxin mAb and B) 1 μg/ml of anti-Sp1 mAb. β-Actin was blotted as a loading control. The experiment was repeated three times.

The role of methylation in controlling the Podxl gene was also investigated studying the effects of a demethylating agent, 5'-aza-2' deoxycitidine (5-azaC), on the podxl expression. Fig. 8A shows that 5-azaC was unable to enhance the expression of podxl in HEK293 cells. In contrast, a 5-azaC-concentration-dependent increase of podxl was observed in Meg0l cells. Treatment with 5-azaC did not modify the Sp1 cellular content (Fig. 8B).

**Figure 9:**
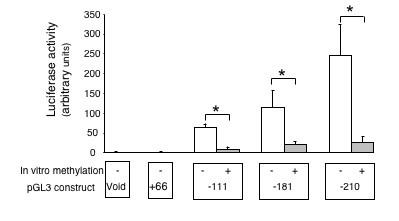
**Effect of *in vitro *DNA methylation on the podocalyxin promoter-driven luciferase expression**. Transient transfections were performed in HEK293 cells. Results represent mean ± SEM from three independent experiments performed in duplicates. Promoter activity of each transfection was expressed as a ratio of luciferase/renilla activities. Data are expressed as arbitrary units calculated as fold increases over void pGL-3 enhancer vector activity, which is assigned a value of 1.0. *p < 0.05 vs. mock methylated control.

To further assess the influence of CpG methylation on the promoter activity of Podxl, we methylated promoter fragments *in vitro *by Sss I followed by their transfection into HEK293 cells. Fig. 9 shows the results of these experiments using either mock methylated or methylated -111,-181 and -210 constructs. The *in vitro *methylation of either construct by Sss I almost abolished the promoter activity.

## Discussion

In this work we have analyzed the structure and function of the Podxl gene promoter. This protein was originally described in the podocytes of the epithelial glomerular cells [1]. Podxl is also present in extra-renal tissues, particularly in the vascular endothelial and hematopoietic cells. Moreover, podxl has also been found in tumor cells [21-23] and its expression has been considered as a good marker of mieloproliferative disorders [24]. The interest on podxl has been recently focused on its putative capacity to regulate the intercellular contacts.

The information about the regulation of the Podxl gene is scarce. The Wilms tumor suppressor factor (WT1) is a potent regulator of kidney induction and nephrogenesis [25,26] that controls the expression of Podxl [14]. However, the WT1 factor is downregulated in all tissues except in the glomerular epithelial cells where it is expressed throughout life. Mutations in the WT1 gene are associated with glomerulosclerosis in patients of Denys Drash syndrome indicating the importance of this factor in the renal function in adulthood. Moreover, nephrogenesis can be rescued in WTl-null mice by reintroducing the gene [26]. However, regulatory factors other than WT1 in renal or extra-renal tissues are undefined. The lack of TATA or CAAT boxes in the Podxl promoter, its high content of C+Gs and the control by Sp1 sites are typical features of house-keeping genes. The transcriptional activity of the progressive 5' deletions of this promoter indicated that its basic activity was located in the region 66 to -111 nts increasing as the length of the DNA fragment increased (Fig. 1). The progressive rise in the promoter activity directly correlates with the number of recognition sites for Sp1, suggesting its functional importance. This interpretation is supported by the absence of transcriptional activity of the Podxl promoter constructs in insect cells (Drosophila SL2) that lack Sp1 transcription factor and the initiation of transcriptional activity by cotransfection of an expression plasmid encoding Sp1 (Fig. 3). Despite differences in endogenous content of podxl, the activity of the Podxl promoter was similar whether Tera-1, HepG2 or HEK293 cells were used in the transfection experiments (Fig. 1). This observation may find an explanation in the similar levels of Sp1 factor found in these cells (Fig. 2).

The nucleotide competition assays gave positive results for Sp1 and AP2 whether nuclear extracts from Tera-1 or from Meg0l cells were used; however, no supershift was observed when monoclonal against AP2 was utilized (Fig. 4B). The competitive effect of AP2 oligonucleotide in the formation of protein-DNA complexes may be related to the sequence similarities between Sp1 and AP2 recognition sites. The EMSA studies indicate that overlapped Sp1 sites -167/-140 nts, -91/-68 nts and -204/-189 nts were able to interact with Sp1, since some of the formed complexes were supershifted by anti-Sp1 Ab and their formation was-prevented by mutating the recognition sites. Moreover, the mutation of the Sp1 sites reduced the transcriptional activity of Podxl.

DNA methylation is associated with malignant transformation [27] of cells but knowledge of the epigenetic events in the initiation and progression of human cancer is limited. The abundant number of CpG islands in the Podxl promoter suggested that methylation could be a regulatory factor. Transcriptional repression of methylated genes may be mediated by the binding of certain proteins, such as MeCP2 [28-30]. Moreover, DNA methylation may interfere with the binding of Sp1 to DNA [31].

Demethylation of the promoter DNA may be a necessary step in the epigenetic reprogramming of somatic cell nuclei [19]. Demethylation is a selective process, operating only on the promoter, but not on enhancers; both a putative Sp1/Sp3 and a GGGAGGG binding site are required for demethylation and the initiation of transcription. We analyzed whether methylation of the Podxl promoter could influence its transcriptional activity: firstly, using demethylating agents in vivo and in second place by studying the state of methylation of the CpG islands in cultured cells expressing different amounts of Podxl. As a demethylating agent we used 5-azaC, structural analog of citidine, that inactivates methyltransferases upon its incorporation into DNA with the result of activation of DNA transcription. This inhibitor has been used successfully as a therapeutic agent in acute myeloid leukemia and myelodysplastic syndrome [32]. In our case, treatment of low podxl-expressing cells with 5-azaC induced a dose-dependent increase on podxl content whereas no effects could be detected in cells, like HEK293, expressing higher levels of podxl. However, the reported effect of 5-azaC in inducing the activity of genes in the absence of methylation of CpG islands indicates that these results should be taken cautiously [33]. The treatment with 5-azaC may stabilize and increase the endogenous content of Sp1 [34] nevertheless in our case 5-azaC did not modify Sp1 cellular content. On the other hand, bisulfite treatment of genomic DNA from high (Tera-1) or low (Meg0l) podxl-expressing cells showed a direct relationship between the degree of methylation of the promoter region and expression of podxl. The influence of the CpG methylation was further assessed by treating the promoter constructs -111,-181 and -210 with the methylase Sss I prior to transfection into HEK293 cells. These results add further evidence indicating that methylation may inhibit the activity of the Podxl promoter.

## Conclusion

The *in vitro *transcription of the human Podxl promoter is dependent on the presence of Sp1 sites. This conclusion is supported by the following observations: 1) No activity was detected when the promoter constructs were transfected into Sp1-lacking insect cells in which the transcription was initiated by cotransfection of an Sp1 expression plasmid; 2) The promoter function was impaired by mutation of the Sp1 sites.

Methylation of the CpG promoter islands seems to be a significant regulatory mechanism in controlling the transcription of Podxl. The latter observation may provide an explanation for the different rates of podxl expression in different tissues of the organism.

## Methods

### Materials

Cell culture media was from GIBCO, Invitrogen (Scotland, UK). Fetal bovine serum was from ICN Biochemicals (Irvine, CA, USA). Restriction endonucleases were obtained from Roche Diagnostics, SL (Barcelona, Spain). Antibody against β-actin was from Sigma RBI (Madrid, Spain); mAbs against Sp-1 (sc-420x and sc-420) or AP2 (sc-25343x) were from Santa Cruz Biotechnology (Santa Cruz, CA, USA). Mouse mAb against human podxl (clone "3D3") was a generous gift of Dr. David B. Kershaw from the Department of Pediatrics, University of Michigan. [α-32P]dCTP was from Amersham Iberica (Spain). The Dual-Luciferase Reporter Assay System, pGL3-basic vector and Wizard DNA Clean-Up System were from Promega (Madison, WI). Sss I (CpG) methylase was from New England Biolabs (Beverly, MA). Oligonucleotides were obtained from Invitrogen (Paisley, UK). All other reagents were of analytical grade and were purchased from commercial sources.

### Construction of reporter plasmids

A 1528-bp DNA fragment was amplified by PCR from human genomic DNA using the primers Podxl-pr -1297/-1275 (5'-GAGCGGGGGAGGGGAAAGGAA-3') and Podxl-pr 231/208 (5'-GGAGCAGGTGGCTGCGGTGC-3') as sense and antisense oligonucleotides, respectively. The PCR product was subcloned directly into a T vector (Topo TA cloning, Invitrogen) to introduce Xho I and Hind III restriction sites in 5' and 3' ends, respectively. The Xho I/Hind III (-1297/231) fragment was subcloned into Xho I/Hind III sites of pGL3-basic to yield the construct -1297. Construct -1234 was produced by partial digestion with EcoR I and religation of the -1297 construct. Constructs -111, -181, -210, -364, -851, and -1108 were obtained by PCR amplification from construct -1297 using as antisense primer Podxl-pr 231/212 (5'-GCCTCGAGCAGGTGGCTGCGGT-3', underlined bp were mutated to generate a Xho I site) and one of the following sense primers:

Podxl-pr -111/-93 (5'-TGGCGAGCTCCTGGGGGAGGG-3'), Podxl-pr -181/-161 (5'-GCTCAGAGCTCACGGCCCAGCG-3'), Podxl-pr -210/-189 (5'-GTCGAGCTCCAGGGCGGAGTTTC-3'), Podxl-pr -364/343 (5'-GGGAGCTCGCCGGGCCCACTTA-3'), Podxl-pr -851/-830 (5'-CTGAGCTCAGAGGCAGGTTTGC-3'), Podxl-pr -1108/-1087 (5'-TTGAGCTCCCCAACAACCCCCT-3'). Underlined nucleotides in sense oligonucleotides were mutated to generate Sac I sites. pGL3-constructs with mutated Sp1 sites were obtained by PCR from DNA template and antisense oligonucleotide mentioned above and the following sense primers: Podxl-pr -90/-79 mutSp1-A (5'-GAGCTCCGGTGGTGGGGGTGGGGG-3'), Podxl-pr -146/-126 mutSp1-B (5'-CAGCTCCGGCCTTGCCCGGCC-3'), Podxl-pr -178/-150 mutSp1-C (5'-TCATTGTTCAAAGCCCAGAGCCCACCCAG-3'), Podxl-pr -207/-184 mutSp1-C (5'-GAGCTCCAGGTTGGAGTTTCC-3'). Underlined nucleotides show mutated nucleotides. The resulting PCR products were subcloned into a T vector and sequenced in an ABI PRISM 3100 Genetic Analyzer (Applied Biosystems, Foster City, CA). The TOPO constructs were digested with Sac I and Xho I and ligated to the Sac I and Xho I sites of pGL3-basic plasmid. Restriction enzyme digestion, sequence analysis, or both were used to verify the constructions. A computer analysis for potential binding sites on DNA sequences was carried out with the TFSEARCH program [19].

### Cell lines

HepG2 (hepatoma cells) and HEK293 (human embryonic kidney cells) were grown in DMEM, Tera-1 (testes embryonal carcinoma cells) in McCoy medium, Meg0l (human megakaryoblastic cells) in RPMI medium, all obtained from GIBCO (Madrid, Spain), and Drosophila Schneider's SL2 cells in Schneider's insect medium (Sigma, Madrid, Spain). Culture media were supplemented with 10% fetal bovine serum. SL2 cells were grown at 25°C and all other cell lines at 37°C.

### Transient transfection procedure

HepG2, Tera-1 and HEK293 cells were transfected by the calcium phosphate precipitation technique in 60-mm dishes with 2 μg of pGL3/Podxl constructs described above and 0.5 μg of phRL-cmv plasmid for transfection efficiency correction. For negative and positive controls of transfection, pGL3-basic and the 3TP promoter-luciferase construct (p3TP-lux), containing three copies of the TPA response element from the human collagenase promoter plasmids were used. SL2 cells were cotransfected with pGL3/Podxl constructs and either pPAC-Sp1 that expresses human Sp1 driven by the *Drosophila *actin promoter, or the control vector pPacO, containing only the *Drosophila *actin promoter. The amount of transfected DNA was maintained constant with void plasmid. Either 6 h (HEK293 and Tera-1 cells) or 16 h (HepG2 and SL2 cells) after transfection, the medium was changed and cells were incubated for another 48 h. The luciferase activity was determined using the Dual-Luciferase Reporter Assay System (Promega, Madison, WI, USA) in cell extracts prepared according to manufacturer recommendations in a TD-20/20 luminometer (Turner Designs, Sunnyvale, CA, USA). Luciferase activities were normalized by Renilla luciferase activity, except for SL2 experiments that were normalized by the protein concentration that was measured using a Bradford protein assay kit (Bio-Rad Laboratories, Inc., Richmond, CA, USA).

### Gel retardation assay

DNA probes were prepared by PCR amplification from the -1296 construct. The following sense and antisense primers were used: for -243/-18 probe: Podxl-pr -243/-223 (5'-CCAAGGCTCGAGGCCGCCGG-3') and Podxl-pr -18/-38 (5'-CACTGGCGGCTGCGGCTACTC-3'); for-538/-238 probe: Podxl-pr-538/-518 (5'-GGGCTCGAGAGGCAGGTGAAT-3') and Podxl-pr -238/-258 (5'-CCTTGGGGGGCTGTGTGTGCG-3'), for A probe: Podxl-pr -111/-93 and Podxl-pr -18/-38, for mutSp1-A probe: Podxl-pr -90/-79 mutSp1-A and Podxl-pr -18/-38, for B probe: Podxl-pr -146/-136 (5'GAGCTCCGGCCCCGCCCGGCC-3') and Podxl-pr -94/-114 (5'CTCGAGCAGGAGCCCGCCAGC-3'); for mutSp1-B probe: Podxl-pr-146/-126 mutSp1-B and Podxl-pr -94/-114; for C probe: Podxl-pr-181/161 and Podxl-pr -94/-114; for mutSp1-C probe: hPodxl-pr -178/-150 mutSp1-C and Podxl-pr -94/-114; for D probe: Podxl-pr -210/-189 and Podxl-pr -150/-178mutSp1-C (5'-CTGGGTTGGTTCTGGGCTTTGAATGA-3'); for mutSp1-D probe: Podxl-pr -207/-184 mutSp1-D and Podxl-pr -150/-178mutSp1-C. All the probes were labeled using the Klenow fragment of Escherichia coli polymerase and [^32^P]dCTP. Nuclear extracts from Tera-1 and Meg0l cells were prepared according to standard protocols. Nuclear extracts (5 μg) were preincubated for 15 min at 4°C in 25 μL-binding reaction containing: 10 mM Tris/HCl pH 7.5, 50 mM NaCl, 2.5 mM MgC12, 4% glycerol, 0.5 mM dithiothreitol and 3 μg poly(dI-dC). After adding ~30 pg of labeled DNA, the mixture was incubated for 30 min at room temperature, and the complexes formed resolved on a 4% nondenaturing polyacrylamide gel in 0.5 × TBE buffer. Gels were run at 150 V at room temperature, dried and exposed at -80°C to an X-ray film.

For competition assays, 100-fold molar excess of unlabeled double stranded DNA consensus sequences of binding sites for AP2, Sp1, NFkB and GATA transcription factors were included in the binding reaction. These DNA fragments were prepared by hybridization of sense and antisense oligonucleotides of the following sequences: 5'-GATCGAACTGACCGCCCGCGGCCC-3' for AP2, 5'-ATTCGATCGGGGCGGGGCGAG-3' for Sp1, 5'-AGTTGAGGGGACTTTCCCAGGC-3' for NFkB and 5'-CACTTGATAACAGAAAGTGATAACTCT-3' for GATA DNA binding sites. As nonspecific competitor, an unrelated DNA fragment of similar size and base composition was used. In supershift experiments, nuclear extracts were preincubated with either mAbs against Sp1, AP2 or nonspecific antibodies, prior to incubation with the radiolabeled probes.

### In Vitro methylation reactions

To examine the effect of CG methylation on the promoter activity, Podxl promoter constructs -111,-181,-210 were mock methylated or methylated by Sss I methylase in the absence or presence of S-adenosylmethionine. The status of methylation was determined by digestion with the restriction enzyme Hpa II. Mock-methylated or Sss I methylated plasmids were isolated from 1% agarose gel using Gel Purification Kit from Qiagen.

### Western blot analysis

For western blot analysis, cells were lysed in 100 μl of phosphate buffered saline containing 1% Triton X-100, 0.1% SDS, 2 mM phenylmethylsulfonyl fluoride, 2 mM EDTA, and 1% of protease inhibitors cocktail. Cellular extracts were resolved by SDS-PAGE and transferred to PVDF membranes. The membranes were incubated with mAb against Sp1 (Sta Cruz sc-420) or Podxl (3D3) and subsequently with the horseradish peroxidase-conjugated secondary antibody (BioRad, Madrid, Spain). β-Actin was blotted in order to normalize the amount of lysate in the experiments. To develop the blots we use the ECL reagent.

### Treatment of cells with 5-Aza-2 deoxycytidine

The cells were treated with 5-azaC at a final concentration of 1 μM or 5 μM for 7 days. The medium was changed every second day. Negative controls were treated in the same manner without 5-azaC. Then, cells were lysed for western blot analysis and blotted with the mAb anti-Podxl primary antibody (3D3).

### Bisulfite modification of genomic DNA

To release small fragments of genomic DNA of Meg0l and Tera-1 cells we digested with restriction enzymes that don't cleave the CpG region of the Podxl-pr. Digested genomic DNAs were denatured, modified by bisulfite, purified, and desulphonated according to Herman et al [35]. Degenerated primers for PCR were designed to amplify the region between -618 to +368 (Podxl-pr -618/-592 5'-GGTGAAGGAGTGAATTAGTC/TC/TAGC/TTTG-3' and Podxl-pr 368/343 5'-AGAATGGTGAGTGGC/TC/TGGC/TC/TTGGGTT-3). The PCR fragments were purified by the JetQuick Purification Kit (Genomed) and sequenced.

## Competing interests

The author(s) declare they have no competing interests.

## Authors' contributions

NB participated in the experimental design, helped to draft the manuscript and carried out experiments shown in Figs. 2 to 6 and collaborated in experiments shown in Figs 1 and 9. SL performed experiments shown in Fig. 5 and contributed to the conceptual basis of the study. SA, RBR and CGM performed the computer analysis of the promoter region and prepared some of the constructs used along this work. EGA-S and MSA participated in the experiments shown in Figs. 3 and 4. RP designed, coordinated and drafted the manuscript. All authors approved the manuscript.
